# RELATIONSHIPS BETWEEN BLOOD BIOENERGETICS AND LEG COMPOSITION ACROSS THE HUMAN LIFE COURSE

**DOI:** 10.1093/geroni/igae098.2299

**Published:** 2024-12-31

**Authors:** Adrian Arciniega, Howard Phang, Jaclyn Bergstrom, Anthony Molina

**Affiliations:** University of California San Diego, La Jolla, California, United States; University of California, San Diego, La Jolla, California, United States; UC San Diego, La Jolla, California, United States; University of California San Diego, La Jolla, California, United States

## Abstract

The human aging process is marked by changes in body composition, such as muscle loss and bone loss, which may be related to mitochondrial dysfunction. Peripheral blood mononuclear cells (PBMCs) are being used to report on systematic mitochondrial function and have been associated with the bioenergetic capacity of various tissues. Given the ability of PBMCs to recapitulate systemic bioenergetics, we investigated the associations between PBMC bioenergetics and lower extremity composition. We hypothesized that PBMC bioenergetics are significantly associated with measures of leg composition. In this study we utilized data from the San Diego Nathan Shock Center (SD-NSC) clinical cohort which measured blood cell bioenergetics and body composition in healthy adults across the human life course. Pearson Correlation analysis (ntotal=48) revealed strong negative correlations between PBMC respiration and leg bone mineral content (basal r=-0.358, max r=-0.274), femoral area (basal r=-0.369, max r=-0.308), and femoral bone mineral content (basal r=-0.405, max r=-0.329). Partial correlation analyses indicated that significant associations persist when controlling for age and BMI. Further stratification by sex revealed that these relationships were primarily driven by female participants (n=29). These results suggest that blood bioenergetics are related to leg composition in adults regardless of age and play a role in body composition, particularly among women.

